# Avian Influenza

**DOI:** 10.3201/eid1508.090095

**Published:** 2009-08

**Authors:** Elizabeth Mumford

**Affiliations:** World Health Organization, Geneva, Switzerland

**Keywords:** Avian influenza, H5N1, viruses, book review

David Swayne has recruited top experts worldwide to contribute to his new book, Avian Influenza. Each of the 25 chapters focuses on a specific area of expertise, and together they offer a variety of perspectives on this disease from the technical to the historical to the strategic.

The history of avian influenza starts with a fascinating look at the domestication of poultry and moves to a description of the first known fowl plague outbreaks in Europe in the late 1800s and a description of the initial determination of the pathogenic agent. Epidemiologic details of outbreaks caused by high and low pathogenicity influenza viruses since that time are given for the different geographic regions. The current panzootic of highly pathogenic avian influenza virus (H5N1) is described event by event since 1996, and the phylogenetic relationships among influenza viruses (H5N1) and potential methods of spread are discussed. This valuable historical and technical background offers context and perspective to ongoing thinking about current (and future) influenza-related events.

Technical chapters cover the epidemiologic and virologic aspects of avian influenza, such as viral determinants of pathogenicity and pathophysiology of different avian influenza viruses in various species of birds and mammals. Also covered are disease control topics such as poultry culling/depopulation, carcass disposal, virus inactivation, biosecurity, vaccinology, and diagnostic testing. Disease impacts on trade and economics, including livelihoods, are also discussed, and a description of the animal health sector strategy for global control of highly pathogenic avian influenza is presented in conclusion. Although much of the recently available research and experience is focused on the H5N1 subtype, Dr Swayne and his collaborators have methodically extended their discussion to include other avian influenza subtypes and both high and low pathogenicity viruses wherever information exists.

The focus of the book is clearly on avian influenza in birds. However, information on public health aspects of the disease is provided in a chapter devoted to this topic as well as in other chapter contexts as appropriate. The relevant aspects of human seasonal influenza and pandemic influenza are reviewed, and the occurrence and impact of zoonotic infections with avian influenza virus (H5N1) and other high and low pathogenicity avian influenza viruses are detailed. In this chapter, the authors emphasize that surveillance and monitoring for avian influenza worldwide should be increased and made more sustainable and that collaborative efforts between animal health and public heath authorities should continue to be strengthened.

The authoritative and comprehensive nature of the information provided, as well as extensive reference lists and a volume-wide index, make this book a valuable reference asset for the scientific community, researchers, veterinary practitioners, governmental animal health agencies, those in the poultry industry, and others working to control and prevent avian influenza globally, including those working in public health. As an added benefit, all book proceeds will go to the American Association of Avian Pathologists to further educational programs on avian diseases worldwide.

